# Measuring the educational environment in a Sri Lankan medical school following curricular revision

**DOI:** 10.1186/s12909-021-02625-8

**Published:** 2021-03-28

**Authors:** Amaya Ellawala, Rohana B. Marasinghe

**Affiliations:** Department of Medical Education, Faculty of Medical Sciences, University of Sri Jayewardenepura, Nugegoda, Sri Lanka

**Keywords:** Curriculum, Education environment

## Abstract

**Background:**

In 2007, the Faculty of Medical Sciences, University of Sri Jayewardenepura revised its medical curriculum from discipline-based to one that was student-centered and integrated. This study aimed to evaluate the perceptions of students regarding the educational environment and compare them to those prior to curricular revision.

**Methods:**

The Dundee Ready Education Environment Measure (DREEM) questionnaire was administered to all volunteering students enrolled in the medical degree programme at the time of the study (*n* = 595). Results were compared to DREEM scores obtained prior to curricular revision.

**Results:**

The overall DREEM score and sub-scale scores were positive and showed improvement compared to previous scores. The score for Students’ Perceptions of Atmosphere showed progression from ‘there are many issues which need changing’ to the next highest category ‘a more positive attitude’. The mean scores in pre-clinical, para-clinical and clinical phases also showed an improvement. ‘The teachers are knowledgeable’ was the highest rated item overall and within each phase of learning. All sub-scales were rated highest by pre-clinical students and lowest by para-clinical students, in contrast to previous results where such patterns were not observed. Certain items, especially those related to teaching/learning, received exclusively low scores in particular student subsets.

**Conclusions:**

Students’ perceptions towards the educational environment overall, have improved following curricular revision. However, certain negative areas warranting further evaluation were highlighted.

## Background

The educational environment encompasses the multitude of elements within the learning experience of an institution. McAleer and Roff [1] described it as the nature of the educational experience, comprising intangible components such as the culture and ambience of a learning establishment. The importance of an environment conducive to learning has long been recognised as a critical requirement for successful training [2]. Educational strategy and curricular design are known to influence the learning environment, with growing evidence to suggest that the educational climate of schools implementing more student-centered strategies, is more favourably rated in comparison to that of more traditional schools [3].

The Faculty of Medical Sciences (FMS), University of Sri Jayewardenepura (USJ), conducts a five-year medical degree programme, admitting 150–200 students (aged 18–21 years at entry, on a 3:2 female to male ratio) annually, based on academic performance at the GCE Advanced Level examination. The programme commenced in 1992 with a traditional discipline-based curriculum, where learning occurred as discrete subjects (eg. Physiology, Pathology etc.) with little integration and application to the clinical context. Over time, certain shortcomings of the curriculum became apparent: overlap among disciplines, *teacher-*centered learning and lack of emphasis on knowledge integration and application. Due to these deficiencies and the global shift towards more innovative and learner-centric education [4, 5], the curriculum was revised to one that was integrated and system-based, and commenced with the November 2007 intake of medical students. Delivery occurred in three phases: Phase I (pre-clinical/first and second years) - basic sciences, Phase II (para-clinical/third and fourth years) – both clinical placements and classroom-based learning related to para-clinical and clinical subjects, and Phase III (clinical/fifth year) comprising purely clinical appointments in major specialties. Early exposure to the clinical application of theory was provided through hospital visits, clinician led case discussions and skills-laboratory based training. Existing English and Information Technology (IT) modules were reinforced. Noteworthy introductions to the curriculum were a Personal and Professional Development Stream (PPDS) to support the professional identity formation of students through learning related to medical professionalism, and a Community Based Medical Learning (CBML) programme where students would spend a week residing in a rural setting and receive exposure to low-resource, primary healthcare institutions. Student-centered learning was promoted by increasing the number of small group discussions, student presentations and group activities, and providing dedicated time for self-learning. Overall, the new curriculum focused on integration and application of knowledge and promoted more *student*-centered learning.

Soemantri, Herrera and Riquelme [6] emphasised the importance of educational environment evaluation as one of the ‘good practices’ of an institution. Moreover, student perceptions of their learning environment are recognised as ‘crucial within the context of learning’ [3] (p1240). On this basis, measurement of the educational environment was considered an essential element of evaluating the revised curriculum. Furthermore, as the educational environment had been appraised prior to curricular transition, there existed valuable opportunity for comparisons to be made with previous results.

The Dundee Ready Education Environment Measure (DREEM) questionnaire, developed by Roff et al. [7] and a Delphi panel of over 100 health professions educators from across the globe, is one of the most widely used tools for measuring the educational environment of medical undergraduate settings [8]. DREEM is culturally non-specific, and has been extensively used around the globe, predominantly in Europe and Asia [3].

At the FMS, USJ it was used to evaluate the educational environment prior to curricular revision, revealing an overall score of 107.7 (educational environment that is more positive than negative) [9]. With the shift to a more modern curriculum, we recognised the possibility of the educational environment having evolved in a positive direction.

DREEM has been described as an effective diagnostic tool in identifying the problem areas plaguing a learning environment [10]. Soemantri, Herrera and Riquelme [6] outlined its ability to differentiate between the climate of traditional versus a more innovative medical school, while Chan et al. [3] emphasised the value of including DREEM scores in longitudinal evaluations of educational environment following curricular interventions. These factors favoured the use of DREEM in the current study.

## Methods

### Study aims

This study was conducted in order to determine the perceptions of students regarding the educational environment of the FMS, USJ following curricular revision, and to compare the results with those of the previous evaluation (henceforth referred to as the ‘past evaluation’), thereby enabling the institution to identify issues requiring attention and make inferences regarding changes brought about with the curricular revision.

### The DREEM questionnaire

DREEM is a 50-item questionnaire that provides a global score out of 200 and comprises items related to five sub-scales: students’ perceptions of learning (SPL), students’ perceptions of teachers (SPT), students’ academic self-perceptions (SAP), students’ perceptions of atmosphere (SPA) and students’ social self-perceptions (SSP). The overall and sub-scale scores are interpreted according to the categories depicted in Table 1.

**Table 1:** Guide to interpretation of overall and sub-scale scores of DREEM

Score		Interpretation (categories)
DREEM score	0–50	Very poor
	51–100	Plenty of problems
	101–150	More positive than negative
	151–200	Excellent
SPL	0–12	Very poor
	13–24	Teaching is viewed negatively
	25–36	A more positive approach
	37–48	Teaching highly thought of
SPT	0–11	Abysmal
	12–22	In need of some retraining
	23–33	Moving in the right direction
	34–44	Model teachers
SAP	0–8	Feeling of total failure
	9–16	Many negative aspects
	17–24	Feeling more on the positive side
	25–32	Confident
SPA	0–12	A terrible environment
	13–24	There are many issues that need changing
	25–36	A more positive atmosphere
	37–48	A good feeling overall
SSP	0–7	Miserable
	8–14	Not a nice place
	15–21	Not too bad
	22–28	Very good socially

*DREEM* Dundee Ready Education Environment Measure, *SPL* students’ perceptions of learning, *SPT* students’ perceptions of teachers, *SAP* students’ academic self-perceptions, *SPA* students’ perceptions of atmosphere, *SSP* students’ social self-perceptions

Responses to items in DREEM are provided on a 5-point Likert scale and subsequently scored from 0 to 4 (strongly agree = 4, agree = 3, unsure = 2, disagree = 1, strongly disagree = 0), except for 9 negatively expressed items (4, 8, 9, 17, 25,35, 39, 49 and 50) which are scored in reverse order. Therefore, the higher the value, the more positive the perceptions towards a particular item. Items that receive scores above 3 are generally considered as positive aspects of the educational environment, while those with scores between 2 and 3 are considered areas that are acceptable but with room for further improvement. Scores below 2 signal negative elements that require intervention.

### Data collection and analysis

This cross-sectional descriptive study was conducted in 2014, seven years after implementation of the integrated curriculum, as the first batch of students to follow the revised medical curriculum completed their training. The DREEM questionnaire was administered to all batches (Phases I, II and III) enrolled for the medical degree programme at the time.

The questionnaires were distributed immediately following a scheduled lecture of each cohort. Therefore, only consenting lesson attendees participated. The first author provided a brief introduction, emphasizing the importance of the exercise following which, 20 min were provided for the questionnaire to be completed and returned. The original English version of DREEM was used, as the students were determined to be adequately proficient in the language to complete this version, given that they followed the medical degree programme entirely in English. However, certain terms that were felt to be ambiguous were explained in the introduction by the first author who additionally provided further clarifications when required.

Data was analysed using SPSS (version 21). DREEM and sub-scale scores were statistically compared between the three phases of learning.

### Past evaluation

The past evaluation, conducted in 2002 (five years prior to curricular revision), included all batches enrolled at the time, with the participation of 339 students (147, 116 and 76 from pre-clinical, para-clinical and clinical stages of training respectively). The data collection process was similar to the one followed in the present study.

## Results

A total of 595 students (164 = pre-clinical, 300 = para-clinical and 131 = clinical) participated in the study. The male:female ratio was 2:3. The gender distribution of the three student subsets were similar.

### Overall DREEM score and sub-scale scores

The overall DREEM score was 124.4, falling into the category ‘more positive than negative’. The mean DREEM scores in the pre-clinical, para-clinical and clinical phases were, 132, 120 and 125 respectively (*p* < 0.05), also falling within the same category. Though the overall score showed improvement from the previous score of 107.7, the category has remained static. Similarly, the mean scores of the three student subsets also remain at the same category level, though the numerical scores have improved (107, 110 and 107 respectively).

The mean scores in the five sub-scales were: SPL = 31.1 (a more positive approach), SPT = 27.5 (moving in the right direction), SAP = 20 (feeling more on the positive side), SPA = 29 (a more positive atmosphere) and SSP = 16.9 (not too bad). Table 2 compares the present and past overall and sub-scale scores in the total student population and student subsets.

**Table 2:** Comparison of past and present overall DREEM score and sub-scale scores in all students and student subsets

	All students		Pre-clinical students		Para-clinical students		Clinical students	
	Past (***n*** = 339)	Present (n = 595)	Past (***n*** = 147)	Present (***n*** = 164)	Past (***n*** = 116)	Present (***n*** = 300)	Past (***n*** = 76)	Present (***n*** = 131)
Overall DREEM score	107.7	124.4	106.5	132.1	109.7	120.1	107.2	125.0
SPL	25.6	31.1	25.9	32.9	25.1	30.1	25.6	31.3
SPT	23.3	27.5	21.8	30.3	25.7	25.9	22.5	27.6
SAP	17.5	20	16.4	20.2	17.8	19.8	19.1	20.2
SPA	24.9	29	25.2	31.1	25.2	27.7	23.6	28.8
SSP	15.2	16.9	15.0	17.5	13.8	16.5	14.8	17.0

*DREEM* Dundee Ready Education Environment Measure, *SPL* students’ perceptions of learning, *SPT* students’ perceptions of teachers, *SAP* students’ academic self-perceptions, *SPA* students’ perceptions of atmosphere, *SSP* students’ social self-perceptions

All sub-scales were rated highest by pre-clinical students and lowest by para-clinical students (*p* < 0.05). This was in contrast to the past evaluation, where such patterns did not emerge. Of the five sub-scales, para-clinical and clinical groups rated SPL as highest. The most favourably rated sub-scale in the pre-clinical group was SPT. SPA was the lowest rated sub-scale among para-clinical and clinical students, while SSP received the lowest score in the pre-clinical group.

**Fig. 1:**
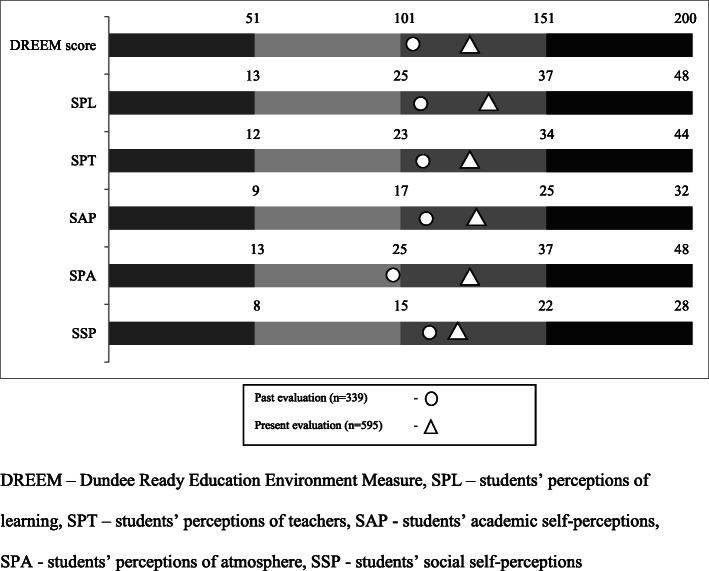
Comparison of past and present overall DREEM and sub-scale scores of entire student population

Figure 1 provides further illustration of the differences in past and present DREEM scores and sub-scale scores, for the overall student population. As depicted, the categories have remained stable for all sub-scales except SPA which has progressed to a category one higher than the previous level.

### Item mean scores

Three items received scores of above 3, compared to a single item in the past evaluation. ‘The teachers are knowledgeable’ received the highest score overall and within each student subset. Incidentally, this was also the highest rated item in the past, but had progressed from a score of 3.26 to 3.52. With a score above 3.5, this can now be considered a ‘strength’ of the institution (Roff et al. 1997). ‘I am encouraged to participate in class’ and ‘I have good friends in this school’ were the other items achieving scores over 3.

Ten items were rated poorly earning scores below 2, in contrast to only eight such items in the past evaluation. ‘The teaching overemphasizes factual learning’ was rated lowest overall with a score of 1.37. The most poorly perceived elements included items such as ‘teachers get angry in class’, ‘I am too tired to enjoy this course’ and ‘teaching is too teacher centered’.

Following curricular revision, an improvement of scores was seen across all items save for twelve, which had declined. Interestingly, these belonged to all sub-scales apart from SSP. Of the twelve items, seven received ratings below 2.

Table 3 provides a comparison of past and present item scores.

**Table 3:** Comparison of past and present overall mean item scores

	Item		Past	Present
SPL	1	*I am encouraged to participate in class*	2.95	3.06
	7	The teaching is often stimulating	2.49	2.85
	13	The teaching is student-centered	2.47	2.78
	16	The teaching is sufficiently concerned to develop my competence	2.81	2.81
	20	The teaching is well focused	2.41	2.87
	22	The teaching is sufficiently concerned to develop my confidence	2.69	2.66
	24	The teaching time is put to good use	2.38	2.70
	25	*The teaching over-emphasizes factual learning*	1.82	1.37
	38	I am clear about the learning objectives of the course	2.45	2.72
	44	The teaching encourages me to be an active learner	2.54	2.71
	47	Long-term learning is emphasized over short-term	2.51	2.62
	48	*The teaching is too teacher-centered*	2.20	1.98
SPT	2	*The teachers are knowledgeable*	3.26	3.52
	6	The teachers are patient with patients	2.52	2.82
	8	*The teachers ridicule the students*	2.15	1.86
	9	The teachers are authoritarian	1.73	2.04
	18	The teachers have good communications skills with patients	2.45	2.92
	29	The teachers are good at providing feedback to students	2.31	2.51
	32	The teachers provide constructive criticism here	2.28	2.51
	37	The teachers give clear examples	2.54	2.86
	39	*The teachers get angry in class*	2.02	1.61
	40	The teachers are well prepared for their class	2.70	2.93
	50	*The students irritate the teachers*	2.27	1.92
SAP	5	Learning strategies which worked for me before continue to work for me now	2.27	2.11
	10	I am confident about my passing this year	2.72	2.66
	21	I feel I am being well prepared for my profession	2.50	2.47
	26	Last year’s work has been a good preparation for this year’s work	2.55	2.57
	27	*I am able to memorize all I need*	1.40	1.63
	31	I have learned a lot about empathy in my profession	2.76	2.95
	41	My problem-solving skills are being well developed here	2.35	2.66
	45	Much of what I have to learn seems relevant to a career in medicine	2.84	2.93
SPA	11	The atmosphere is relaxed during the ward teaching	1.56	2.21
	12	This school is well time-tabled	2.19	2.65
	17	*Cheating is a problem in this school*	2.21	1.90
	23	The atmosphere is relaxed during the lectures	2.47	2.71
	30	There are opportunities for me to develop inter-personal skills	2.47	2.86
	33	I feel comfortable in class socially	2.56	2.78
	34	The atmosphere is relaxed during seminars/tutorials	2.18	2.35
	35	*I find the experience disappointing*	1.90	1.84
	36	I am able to concentrate well	2.36	2.51
	42	The enjoyment outweighs the stress of studying medicine	2.13	2.12
	43	The atmosphere motivates me as a learner	2.29	2.54
	49	I feel able to ask the questions I want	2.16	2.45
SSP	3	There is a good support system for students who get stressed	1.52	2.27
	4	*I am too tired to enjoy this course*	1.35	1.66
	14	*I am rarely bored on this course*	1.92	1.96
	15	*I have good friends in this school*	2.79	3.05
	19	My social life is good	2.72	2.79
	28	I seldom feel lonely	2.09	2.25
	46	My accommodation is pleasant	2.62	2.91

*SPL* students’ perceptions of learning, *SPT* students’ perceptions of teachers, *SAP* students’ academic self-perceptions, *SPA* students’ perceptions of atmosphere, *SSP* students’ social self-perceptions

Items in which scores have declined following curricular revision are shaded

Items that have received scores above 3 or below 2 in the present evaluation, are italicised

Item scores were further analysed according to the phase of learning (Table 4). The highest frequency of scores above 3 emerged in the pre-clinical group, while at the other extreme of the spectrum, the clinical group recorded the most items with scores below 2. ‘I am encouraged to participate in class’ and ‘the teachers are knowledgeable’ received high ratings from all three student subsets. Several items were collectively rated poorly, including the lowest rated item overall, ‘the teaching over-emphasizes factual learning’. Others such as ‘learning strategies which worked for me before continue to work for me now’, ‘the students irritate the teachers’ and ‘the teachers are authoritarian’, appeared to be problems specific to the pre-clinical, para-clinical and clinical groups respectively.

**Table 4:** Mean item scores in pre-clinical, para-clinical and clinical student subsets

Sub-scale	Item	Pre-clinical	Para-clinical	Clinical
**Scores > 3**				
SPL	I am encouraged to participate in class	3.06	3.07	3.04
SPT	The teachers are knowledgeable	3.58	3.46	3.59
SPT	The teachers have good communications skills with patients			3.04
SPT	The teachers give clear examples	3.05		
SPT	The teachers are well prepared for their class	3.18		
SAP	Much of what I have to learn seems relevant to a career in medicine	3.15		3.02
SPA	This school is well time-tabled	3.09		
SPA	I feel comfortable in class socially	3.19		
SSP	I have good friends in this school	3.24		3.24
**Scores < 2**				
SPL	The teaching over-emphasizes factual learning	1.44	1.35	1.33
SPL	The teaching is too teacher-centered		1.62	
SPT	The teachers ridicule the students		1.37	1.92
SPT	The teachers are authoritarian			1.59
SPT	The teachers get angry in class		1.28	1.66
SPT	The students irritate the teachers		1.56	
SAP	Learning strategies which worked for me before continue to work for me now	1.98		
SAP	I am able to memorize all I need	1.45	1.75	1.59
SPA	Cheating is a problem in this school		1.52	1.99
SPA	I find the experience disappointing		1.60	1.98
SPA	The enjoyment outweighs the stress of studying medicine	1.99		1.91
SSP	I am too tired to enjoy this course	1.75	1.57	1.75
SSP	I am rarely bored on this course	1.87		1.95

*SPL* students’ perceptions of learning, *SPT* students’ perceptions of teachers, *SAP* students’ academic self-perceptions, *SPA* students’ perceptions of atmosphere, *SSP* students’ social self-perceptions

## Discussion

### Overall DREEM score and sub-scale scores

The present DREEM score of the FMS/USJ is 128, an improvement from the previous score of 107.7, placing the institution on the second highest level of the DREEM score scale: ‘more positive than negative’. This improvement is in keeping with the expectation that the learning environment would improve following the shift from a teacher-led to a more student-centric educational system [10, 11], and additionally coincides with the overwhelming majority of studies that report DREEM scores falling within the same range [3].

It is encouraging to note that the overall ratings in each subscale too have improved. Aside from SPA, scores of the other four sub-scales remain stable, though having advanced numerically within each category. SPA on the other hand, has progressed to the next category, indicating a more positive attitude of students towards the atmosphere of the institution. With the revision of the curriculum much emphasis was placed on promoting fellowship and building interpersonal relations among students, especially during the Orientation Programme and within the PPDS. It is possible that these measures may have contributed to favourable perceptions regarding the atmosphere. However, the opinion that the atmosphere in formal learning settings is conducive sits in stark contrast to the finding that students appear to irritate and anger teachers. These conflicting results raise the question that respondents may not have accurately understood what ‘atmosphere’ in the learning environment encompassed.

It is promising to note that perceptions towards student support systems have improved overall, possibly as a result of strengthened mentoring schemes, peer-support systems and counselling services. This diverges from the literature where problems surrounding psychosocial support appear to be a recurring theme [12, 13].

An intriguing finding is that all sub-scales have received the highest ratings from pre-clinical and the lowest scores from para-clinical students, a difference which is statistically significant. These findings are consistent with settings such as UK and Saudi Arabia, where more favourable ratings in the early years have been attributed to the higher motivation of keen new students who are still exploring their educational environment [14, 15]. This presumption may resonate with our setting, where feedback over the years suggests that pre-clinical students perceive their learning experience more positively than their seniors. Yoo and Kim [16] however, argue that there exists no universal tendency in changes of student perceptions towards the educational environment as the course progresses.

Pre-clinical students have rated SPT as highest among the five sub-scales. This is supported by anecdotal evidence which suggests that first and second year students hold the academic staff in very high esteem. The lowest rated sub-scale in this group is SSP, shedding light on the poor inclination of this group towards participating in extra-curricular and social activities. Malaysia and Thailand also report similar findings, where students in the early years recorded low social perceptions [17, 18]. In Sri Lanka, poor social participation could largely be due to the stress of adapting to the novel concept of student-centred learning, having just emerged from a teacher-centric educational system. Additionally, these students who have followed their entire primary and secondary education in either Sinhala or Tamil, face the pressure of adopting English as the primary language of instruction.

Para-clinical and clinical students have scored the sub-scales in a similar manner, rating SPL as the highest and SPA as the lowest. In terms of perceptions towards learning, it has been observed that by the third year, students have adapted to educational methods and have a better grasp of the English language. By this phase, they are also introduced to workplace-based learning, which generally receives positive feedback from students. However, the immense workload during the second and third phases, in addition to often very stressful learning environments in the workplace, may have resulted in such low perceptions of the atmosphere. Similar findings were reported in Malaysia, where poor perceptions towards the atmosphere in the latter stages of training were attributed to the excessive workload [17].

### Item scores

It is encouraging to note that three items have received high scores above 3, in comparison to a single item previously. ‘Teachers are knowledgeable’ has emerged as a strength of the institution similar to many other schools in the region [13, 18, 19]. It is of additional merit that ‘I am encouraged to participate in class’, is in keeping with the student-centred learning approach that is currently promoted.

Twelve items show a declining trend following curricular revision. These items belong to all sub-scales other than SSP; hence it is reassuring to find that as a whole, students’ perceptions regarding their social environment have not deteriorated. Among items that have regressed, the largest decline is seen for ‘teaching over-emphasises factual learning’, which in addition, has received the lowest overall score. It is a cause for concern, that an item that received a low score previously (1.82), has declined further following revision to a curriculum that attempts to *discourage* factual learning. In contrast, perceptions regarding item 4 (*I am too tired to enjoy this* course), which received the lowest score in the past evaluation*,* have improved, though the present score remains below 2. This albeit minor improvement is in keeping with the conscious effort that was made during curricular revision to reduce content overload.

The ten items that have received overall scores below 2, appear to belong to common themes: teacher-centered teaching strategy (items 25, 48), negative attitudes of teachers (items 8, 39, 50), poor perceptions regarding the overall educational experience (items 4, 14, 27, 35) and cheating at examinations (item 17). Cheating appears to be an offense not unfamiliar to medical undergraduates, with reports of it quite prevalent in medical education literature [20]. Similarly, this problem was repeatedly brought to the attention of the academic staff in our institution, resulting in a recent strengthening of measures to curb cheating.

It is a definite cause for concern that students perceive a curriculum that attempts to promote student centered learning, as achieving the opposite. However, this contradictory view towards student-centered education is echoed in other settings, as too is the perception of teachers being angered and irritated by students [13, 17]. Among these broad areas however, ‘teaching is too teacher centered’ and ‘students irritate the teachers’ received unsatisfactory ratings only in the para-clinical group, signalling a problem that may be confined to Phase II of the curriculum.

Similarly, a problem that seems to be restricted to the pre-clinical group is that learning strategies which worked before do not continue to do so. This issue has been repeatedly highlighted by students, who undergo a major transition when progressing from the teacher-centered education of secondary school to the student-centered learning environment of tertiary education. Ogun et al. [21] have described similar findings and argue that the younger students of a learner-centered curriculum maybe less receptive towards the learning environment and the greater responsibility they must shoulder for their own education, due to their idealistic expectations of higher education. Many efforts have been made to ease this transition and empower students to take a greater onus for their own learning. It appears though that such strategies may have to be re-analysed. Nonetheless, it is encouraging to note that they perceive the material that is learnt in the pre-clinical years as being relevant to their future practice, as great efforts were made in the curricular revision to achieve this. Other schools following non-traditional curricula, report similar findings [17].

The perception of teachers as authoritarian appears to be a problem confined to the clinical phase. This too appears to be a common problem faced by many schools offering student-centered curricula [13, 17, 18]. Intriguingly, these findings appear to emerge predominantly from Asian settings, raising the question that the perception of teachers as authoritarian may resonate with the hierarchical disposition of such cultures [22].

Studies that have made comparisons between differing educational strategies have reported lower DREEM and sub-scale scores in traditional schools than in their innovative counterparts [23]. The results of this study reveal a similar trend and therefore reflect the change from a teacher-centered to a learner-centric curriculum. Certain elements however, conflict with this overall picture, conveying the notion that not all expected outcomes of curricular revision may have been achieved. Ogun et al. [21] ventured the explanation that this may be due to a slower evolution of the hidden curriculum, thereby masking the gains of the more innovative formal curriculum. This theory highlights the importance of harmonising all aspects of the curriculum to reach the desired objective.

We understand that in order to assimilate an accurate picture of the educational environment, the voices of the many stakeholders of that environment must be heard. We therefore acknowledge that the views of students alone may not offer a holistic view of the reality. This is one limitation of the study. Another was the inability to make statistical comparisons between the data gathered in the two separate evaluations of the educational environment, as the raw data of the previous study had been destroyed in line with research governance guidelines.

## Conclusion

Students’ perceptions towards the educational environment overall, have improved following curricular revision at FMS/USJ. Evaluation of the educational environment has revealed pertinent information regarding the strengths of the institution as well as shortcomings that are in conflict with the present focus of the curriculum. The evaluation, therefore has served as a preliminary means of identifying areas for improvement thereby paving way for more focused exploration of means to address them.

## Data Availability

The data supporting the findings of this study are available from the corresponding author (AE), upon request.

## Abbreviations

FMS: Faculty of Medical Sciences
USJ: University of Sri Jayewardenepura
PPDS: Personal and Professional Development Stream
CBML: Community Based Medical Learning
DREEM: Dundee Ready Education Environment Measure
